# Reports of Expired Live Attenuated Influenza Vaccine Being Administered — United States, 2007–2014

**Published:** 2014-09-05

**Authors:** Penina Haber, Christopher P. Schembri, Paige Lewis, Beth Hibbs, Tom Shimabukuro

**Affiliations:** 1 Immunization Safety Office, Division of Healthcare Quality and Promotion, National Center for Emerging and Zoonotic Infectious Diseases, CDC

Annual influenza vaccination is recommended for all persons aged ≥6 months (1). Two vaccine types are approved in the United States, injectable inactivated influenza vaccine (IIV) and live attenuated influenza vaccine (LAIV), which is administered intranasally (1). Influenza vaccine typicaly becomes widely available beginning in late summer or early fall. IIV has a standard expiration date of June 30 for any given influenza season (July 1 through June 30 of the following year). In contrast, after release for distribution, LAIV generally has an 18-week shelf life (Christopher Ambrose, MedImmune, personal communication, 2014). Because of its relatively short shelf life, LAIV might be more likely than IIV to be administered after its expiration date. To assess that hypothesis, CDC analyzed reports to the Vaccine Adverse Event Reporting System (VAERS) (2) of expired LAIV administered during July 1, 2007, through June 30, 2014.

Of the 4,699 LAIV reports, 866 (18.4%) involved administration of expired vaccine; 97.5% of these reports did not document any adverse health event. In 95.1% of expired LAIV reports, vaccination occurred after the first week in November, which is approximately 18 weeks from July 1. Historically, by early November, most vaccine has been administered for the season (3). In contrast, of the 49,695 IIV reports, only 96 (0.02%) involved administration of expired vaccine. VAERS is a national, passive surveillance system that accepts reports from anyone (including vaccine recipients, providers, and manufacturers); because of this, it is not possible to definitively conclude that LAIV is more likely to be administered after its expiration date. However, the magnitude of disproportional reporting for this error in expired LAIV use compared with IIV supports the hypothesis.

As a passive surveillance system, VAERS likely captures only a small fraction of expired LAIV administered, so this error might be more common than VAERS data indicate. Most reports had a vaccination date in November or later. Health care providers need to be aware of the short shelf life of LAIV and implement measures to avoid administering expired LAIV, especially from November and onward, when this error appears to be more common. Although the data do not indicate that administration of expired LAIV poses a health risk, revaccination with a valid dose is advised (4). Replacement options for expired LAIV are available at http://www.flumistquadrivalent.com/hcp/ordering_and_returns.html.
