# Erratum

**Published:** 2014-09-12

## Vol. 63, No. 35

In the report, “Notes from the Field: Reports of Expired Live Attenuated Influenza Vaccine Being Administered—United States, 2007–2014,” the third sentence of the first paragraph should read, “Influenza vaccine **typically** becomes widely available beginning in late summer or early fall.”

